# A Modified Memetic Algorithm with an Application to Gene Selection in a Sheep Body Weight Study

**DOI:** 10.3390/ani12020201

**Published:** 2022-01-15

**Authors:** Maoxuan Miao, Jinran Wu, Fengjing Cai, You-Gan Wang

**Affiliations:** 1 College of Mathematics and Physics, Wenzhou University, Wenzhou 325035, China; 194611073127@stu.wzu.edu.cn; 2 School of Mathematical Sciences, Queensland University of Technology, Brisbane 4001, Australia; j73.wu@qut.edu.au (J.W.); you-gan.wang@qut.edu.au (Y.-G.W.)

**Keywords:** gene selection, sheep weight, memetic algorithm, modifications, local search operator

## Abstract

**Simple Summary:**

Due to lacking exploitation capability, traditional genetic algorithm cannot accurately identify the minimal best gene subset. Thus, the improved splicing method is introduced into a genetic algorithm to enhance exploitation capability for achieving balance between exploitation and exploration of GA. It can effectively identify true gene subsets with high probability. Furthermore, a dataset of the body weight of Hu sheep has been used to show that the proposed method can obtain a better minimal subset of genes with a few iterations, compared with all considered algorithms including genetic algorithm and adaptive best-subset selection algorithm.

**Abstract:**

Selecting the minimal best subset out of a huge number of factors for influencing the response is a fundamental and very challenging NP-hard problem because the presence of many redundant genes results in over-fitting easily while missing an important gene can more detrimental impact on predictions, and computation is prohibitive for exhaust search. We propose a modified memetic algorithm (MA) based on an improved splicing method to overcome the problems in the traditional genetic algorithm exploitation capability and dimension reduction in the predictor variables. The new algorithm accelerates the search in identifying the minimal best subset of genes by incorporating it into the new local search operator and hence improving the splicing method. The improvement is also due to another two novel aspects: (a) updating subsets of genes iteratively until the no more reduction in the loss function by splicing and increasing the probability of selecting the true subsets of genes; and (b) introducing *add* and *del* operators based on backward sacrifice into the splicing method to limit the size of gene subsets. Additionally, according to the experimental results, our proposed optimizer can obtain a better minimal subset of genes with a few iterations, compared with all considered algorithms. Moreover, the mutation operator is replaced by it to enhance exploitation capability and initial individuals are improved by it to enhance efficiency of search. A dataset of the body weight of Hu sheep was used to evaluate the superiority of the modified MA against the genetic algorithm. According to our experimental results, our proposed optimizer can obtain a better minimal subset of genes with a few iterations, compared with all considered algorithms including the most advanced adaptive best-subset selection algorithm.

## 1. Introduction

In data mining, feature selection is a fundamental strategy to handle “the curse of dimensionality” [1]. With an effective feature selection procedure, the redundant and irrelevant features are eliminated to improve the performance of the learning process [2,3]. Further, the feature selection approaches can identify small subsets of biologically important genes, which are the most relevant to the target trait, such as genetic diseases and angiotensin-converting enzyme 2 [4,5]. Thus, in this paper, considering a new application to gene selection in a sheep body weight study, we propose a modified memetic algorithm to effectively select the most important genes from more than 52,000 genes.

The filter algorithm and hybrid feature selection algorithm are the main feature selection methods. The filter algorithm is based on data characteristics, such as distance [6], correlation [7], and statistical distribution [8], to select subsets of genes [9]. Although gene selection using filter algorithms is fast and simple, the top k genes contain some redundant and irrelevant genes for not considering correlation between genes and unreliable feature evaluation principle. The hybrid feature selection method is usually utilized to select a few important genes out of a huge number of genes [10]. In the hybrid algorithm, the filter algorithm is firstly utilized to eliminate many genes, then the wrapper algorithm is used to further compact the selected subset of genes [11]. It is worthwhile to note that the filter algorithm may eliminate many useful genes in initial step, and the wrapper method can learn subsets of gene interact with learning algorithm [12]. One of the most typical methods is the hybrid dragonfly black hole algorithm for gene selection for the RNA-seq COVID-19 data, and the authors achieved a good performance for the investigated data [4]. However, the algorithm usually encounters nest effect and produces a sub-optimal subset. Different from two above-mentioned types of feature-selection methods, heuristic methods can effectively overcome nest effect [13,14,15]. Genetic algorithm (GA) is one of heuristic methods that can widely be applied to gene selection [16], which effectively searches the entire gene subset space by combining exploration with exploitation [17]. Exploration can provide a promising subset of genes of the entire gene subset space, while exploitation can guarantee that the promising subset of genes move toward to the best subset of genes. However, GA lacks exploitation capability [18]; this means that GA cannot obtain the core subset of genes. Therefore, local search is incorporated into GA to enhance exploitation capability, and Memetic Algorithm (MA) is proposed [19].

MA is an improvement of GA, which can undergo self-improvement [20]. Currently, various local search operators have been incorporated into GA to improve exploitation capability. For example, the filter algorithm is embedded into GA for fast identification of the important subset of features [21]. The great deluge algorithm is combined with GA to improve fine-tuning capability of GA [22]. Lamarckian learning is incorporated into GA to utilize the most appropriate local search method among local search methods [23]. Moreover, a hybrid method based on dynamic GA and random forest was developed by Pashaei and Pashaei [5] to distinguish a small meaningful set of genes for cancer classification. They have been proven to outperform other state-of-the art feature selection methods, such as filter and heuristic search algorithms, but they cannot maximize exploitation capability as the local search operator is utilized to obtain the best subset of genes as possible. Splicing method [24] can recover the true subset of genes with high probability but cannot limit the size of gene subsets. Therefore, in our present work, a new local search operator together with an improved splicing method, is embedded into GA, which is known as GA based on an improved splicing method, for significant single-nucleotide polymorphisms (SNPs) identification. There exist some advantages in the proposed method: (1) Compared with filter algorithm and hybrid algorithm, it can effectively explore the entire space of gene subsets and find minimal best gene subsets; (2) Compared with GA, it can enhance exploitation capability of GA for achieving balance between exploitation and exploration of GA in term of improved splicing method; and (3) Compared with traditional MA, it can recover the true subset of genes with high probability and limit the size of gene subsets.

The three main contributions in the paper can be given as follows: (1) A new local search operator along with an improved splicing method, is proposed in the paper. The add and del operators are embedded into the splicing method. It can recover the true subset of genes with high probability, and limit the sizes of subsets of genes; (2) A modified memetic algorithm, GA based on an improved splicing method, is proposed in this paper. It can accelerate search to identify the minimal best subset of genes. The improved splicing method is utilized to improve starting points to enhance efficiency of search. The improved splicing method replaces the mutation operator to enhance exploitation capability for achieving balance between exploitation and exploration of GA; and (3) In projects where identification of SNPs for body weights is required, the GA based on an improved splicing method can find the minimal best subset of genes compared with other heuristic methods, including GA [25], β-hill climbing [26], salp swarm algorithm [27], artificial bee colony algorithm [28], sine cosine optimization algorithm [29], and binary particle swarm optimization [30], and adaptive best-subset selection from Zhu et al. [24].

## 2. Methods

In this section, a modified memetic algorithm is proposed by combining an improved splicing method with genetic algorithm. In other words, the improved splicing method is incorporated into GA to accelerate the search for identifying the minimal best subset of genes. The main advantages of the proposed method are two-fold: (1) it can provide the promising subset of genes of the whole gene subset space based on selection and crossover operators; and (2) compared with traditional GA, it has strong exploitation capability and recovers the minimal best subset of genes with high probability based on an improved splicing method.

### 2.1. The Genetic Algorithm

Genetic algorithm is a heuristic algorithm, which is mainly based on selection and crossover operators [31]. Selection operator directs GA to find the most promising subset of genes of the entire gene subset space. The crossover operator has an exploration capability, which can direct GA to escape from sub-optimal locations. The crossover operator, individual representation, initialization, and selection operator are discussed below.

**a. Individual Representation:**

For feature selection, each individual is represented as a subset of genes. Each individual is encoded by a binary vector, i.e., $a_{i}\left(t\right)=\left[f_{1},f_{2},\cdots ,f_{q}\right]$, $f_{k}\in \left\{0,1\right\}$, where $f_{k}=0$ denoted as the *k*-th gene is not selected while $f_{k}=1$ denoted as the *k*-th gene is selected.

**b. Initialization:**

*N* individuals are randomly generated, which consist of a population P(0), i.e., $P\left(0\right)=[a_{1}\left(0\right),\cdots ,a_{i}\left(0\right),\cdots ,a_{N}\left(0\right)]$. The procedure of randomly generating an individual $a_{i}\left(t\right)$ is shown in Algorithm 1. An initial probability $P_{initial}=s/q$ is defined as a gene $f_{i}=1$, where *s* is the expected number of a subset of genes. A gene is randomly assigned as 0 or 1 by the following way: $f_{i}=1$ if $U\left(0,1\right)<P_{initial}$; else, $f_{i}=0$. The entire procedure is repeated until *q* genes are all assigned.
**Algorithm 1** Initialization of individual      1:**Input:** individual $a_{i}\left(t\right)=\left[\right]$, probability $P_{initial}$      2:**for** i=1,2,⋯,q **do**      3:    **if** $U\left(0,1\right)<P_{initial}$ **then**      4:        $a_{i}\left(t\right)=\left[1,a_{i}\left(t\right)\right]$      5:    **else**      6:        $a_{i}\left(t\right)=\left[0,a_{i}\left(t\right)\right]$      7:    **end if**      8:**end for**      9:**Output:** individual $a_{i}\left(t\right)$

**c. Selection Operator:**

Generally, the higher the quality of gene subsets, the more likely they are within the most promising region of entire search space. Therefore, the survival probability of high quality gene subsets should be set higher. Based on this principle [32], a proportional roulette wheel selection is used in the paper. Since error indicators are considered as fitness function in the paper, $F_{i}$ is lower and the individual $a_{i}\left(t\right)$ is more likely to survive. The survival probability is formulated as,

(1) $$P_{i}=\frac{\frac{1}{F_{i}}}{\sum_{i=1}^{N}\frac{1}{F_{i}}},$$

where $F_{i}$ is the fitness value of the *i*-th individual $a_{i}\left(t\right)$. Each subset of genes is allocated with a survival probability. Then, roulette wheel sampling-based survival probability is utilized to select individuals $a_{i}\left(t\right)$.

**d. Crossover Operator:**

The crossover operator has two distinct characteristics [33]: (1) Genes common to parents are preserved in the offspring. It means that some important genes common to parents improved by the improved splicing method can still be retained to the next generation; (2) Produces offspring that are contained in a region of the search space spanned by parents. It means that the produced offspring remain in the promising area when the parents are located in the promising region of the entire search space. The uniform crossover operator is a general form of single-point or two-point crossover, which is used in the paper. It can be flexible to adjust disruptive effect in term of crossover disruption probability $P_{0}$, which is defined as using the bit value of the first parents [34].

**e. The Improved Splicing Method:**

**Figure 1:**
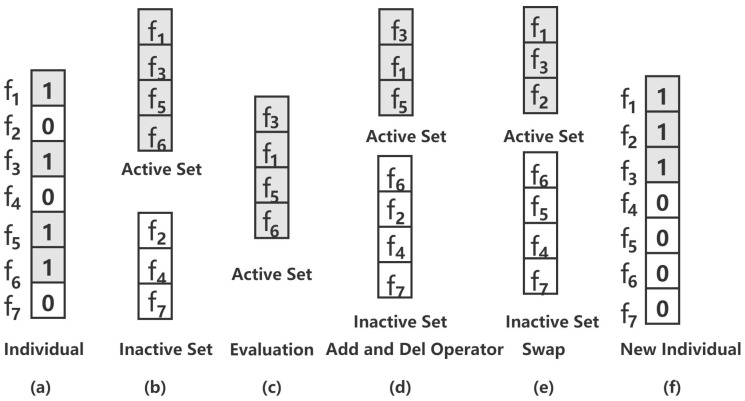
Example of the improved splicing method (**a**) Individual. (**b**) Individual Segmentation. The individuals are divided into active sets and inactive sets. (**c**) Evaluation. The score of each gene in an active set is evaluated in terms of backward sacrifice. (**d**) add and del Operators. Some genes with the lowest scores in active sets are deleted, then added into inactive sets. (**e**) Swap. A gene with the lowest score ($f_{5}$) in an active set and a gene with the highest score ($f_{2}$) in an inactive set swap each other. (**f**) Merge. The active set and inactive set are merged into a new individual.

Splicing method [24], also called the polynomial method, is where subsets of genes can be updated iteratively until the loss function cannot be improved by splicing. It can guarantee separation of unimportant genes from a subset of genes. However, the size of subsets of genes gradually increases over generations. It leads to the minimal best gene subset, which, potentially, cannot be found in higher generations. Therefore, we propose an improved splicing method in this paper. The advantages have three points, as follows: (1) The add and del operators are introduced into the splicing method to limit the maximal size of subsets of genes $S_{max}$. The del operator is used to delete some insignificant genes in active sets to achieve the desirable maximal size of subsets of genes, while the add operator is used to add deleted genes for del operator into inactive sets; (2) It can recover the true subset of genes with high probability; (3) It has strong exploitation capability. Since the mutation operator has less exploitation capability and may introduce some unimportant or irrelevant genes into the subset of genes, mutation operator is replaced by the improved splicing method. The entire process of the improved splicing method mainly is consisted of five parts, i.e., individual segmentation, evaluation, add and del operators, swap, and merge. We illustrate the details of the improved splicing method with an example, as shown in Figure 1, and the algorithm is shown in Algorithm 2.

**Step 1 Individual Segmentation:**

The individual, i.e., $\left[f_{1},f_{2},\cdots ,f_{q}\right]$, is divided into active set $A=\{f_{i}|f_{i}=1,i=1,\cdots ,q\}$ and inactive set $I=\{f_{i}|f_{i}=0,i=1,\cdots ,q\}$. For example, the individual is represented as [1,0,1,0,1,1,0], shown in Figure 1a. It is divided into active set $A=\{f_{1},f_{3},f_{5},f_{6}\}$ and inactive set $I=\{f_{2},f_{4},f_{7}\}$, as shown in Figure 1b.

**Step 2 Judge:**

If the size of active set A is greater than the maximal size of the subset of genes $S_{max}$, go into **Step 3**; otherwise, go into **Step 5**.

**Step 3 Evaluation:**

The backward sacrifice is used to evaluate the score of each gene in active set A. The score of each gene is evaluated by

(2) $$\xi_{j}=\frac{X_{j}^{T}X_{j}}{2n}{\left(\beta_{j}\right)}^{2},$$

where *n* is the number of samples and $\beta_{j}$ is coefficient of the *j*-th gene. In Figure 1c, score of the gene $f_{3}$ is the highest and score of the gene $f_{6}$ is the lowest.

**Step 4 add and del Operators:**

Some genes with the lowest score in active set A are deleted, and then added into inactive set I. The del operator is used to delete $\left|A\right|-S_{max}$ genes with the lowest score in active set A. Deleted $\left|A\right|-S_{max}$ genes are added into inactive set I for add operator. In Figure 1d, $S_{max}$ is set as 3. Since the score of the gene $f_{6}$ is the lowest, the gene $f_{6}$ in active set A is deleted, and then added into inactive set I.

**Step 5 Evaluation:**

The score of each gene in active set A is calculated by backward sacrifice and the score of each gene in inactive set I is calculated by forward sacrifice. Backward sacrifice is formulated as Equation (2), while forward sacrifice is formulated as,

(3) $$\zeta_{j}=\frac{X_{j}^{T}X_{j}}{2n}{\left(\frac{d_{j}}{X_{j}^{T}X_{j}/n}\right)}^{2},$$

where *n* is the number of samples and $d_{j}=X_{j}^{T}\left(Y-X_{A}\beta_{A}\right)/n.$ They are both filter methods based on change in linear loss function. The larger the change in loss function, the more significant the gene is.

**Step 6 Swap:**

*k* genes of the lowest scores in active set A are consisted of set $A_{k}$; *k* genes of the highest scores in inactive set I are consisted of set $I_{k}$. Then, sets $A_{k}$ and $I_{k}$ have swapped each other. Parameter *k* is less than or equal min(|A|,|I|). In order to find the best minimal subset of genes, we should search the optimal parameter *k* from range {1,2,⋯,min(|A|,|I|)} by using grid search. For example, in Figure 1, the parameter *k* is 1. The score of the gene $f_{5}$ in active sets is the lowest and the score of the gene $f_{2}$ in inactive sets is the highest in terms of **Step 5**. Therefore, the sets $A_{k}=\left\{f_{5}\right\}$ and $I_{k}=\left\{f_{2}\right\}$ are obtained. Then, the sets $A_{k}$ and $I_{k}$ have swapped with each other. Finally, the new active set $\left\{f_{1},f_{2},f_{3}\right\}$ and inactive set $\left\{f_{4},f_{5},f_{6},f_{7}\right\}$ are obtained, as shown in Figure 1e.

**Step 7 Update:**

The active set A is updated by repeating **Steps 5–6** until the loss function $L=\frac{1}{2n}||Y-X_{A}\beta_{A}{\left|\right|}_{2}^{2}$ cannot be improved. Then, go to **Step 8**.

**Step 8 Merge:**

The updated active set A and inactive set I are merged to form a new individual, as shown in Figure 1f.
**Algorithm 2** Improved splicing method      1:**Input:** An individual *A*, i.e., $A=[f_{1},f_{2},\cdots ,f_{p}]$, $f_{j}\in \left\{0,1\right\}$, Sample X∈n×p, Y∈n×1, threshold τ; Maximal size of the subset of genes $S_{max}$;      2:The individual *A* is divided into active set $A=\{f_{i}|f_{i}=1,i=1,\cdots ,p\}$ and inactive set $I=\{f_{i}|f_{i}=0,i=1,\cdots ,p\}$;      3:**if **$|A|>S_{max}$ **then**      4:    Calculate the score of each gene in active set A in terms of backward sacrifice.      5:    Delete $\left|A\right|-S_{max}$ genes of the lowest score in active set A to obtain a new active set A; Then deleted $\left|A\right|-S_{max}$ genes are added into inactive set I to obtain a new inactive set I.      6:    $\beta_{A}={\left(X_{A}^{T}X_{A}\right)}^{-1}X_{A}^{T}Y$, $d_{I}=X_{I}^{T}\left(Y-X_{A}\beta_{A}\right)/n$, where $X_{A}\in n\times \left|A\right|$, $X_{I}\in n\times \left|I\right|$;      7:**else**      8:    $\beta_{A}={\left(X_{A}^{T}X_{A}\right)}^{-1}X_{A}^{T}Y$, $d_{I}=X_{I}^{T}\left(Y-X_{A}\beta_{A}\right)/n$, where $X_{A}\in n\times \left|A\right|$, $X_{I}\in n\times \left|I\right|$;      9:**end if**      10:**repeat**      11:    Calculate Loss function $L=L_{0}=\frac{1}{2n}||Y-X_{A}\beta_{A}{\left|\right|}_{2}^{2}$;      12:    Calculate the score $\xi_{j}$ of each gene in active set A in terms of backward sacrifice.      13:    Calculate the score of each gene $\zeta_{j}$ in inactive set I in terms of forward sacrifice.      14:    **for** k=1,2,⋯,min(|A|,|I|) **do**      15:       $A_{k}=\left\{j\in A:\sum_{i\in A}I\left(\xi_{j}\geq \xi_{i}\right)\leq k\right\}$, $I_{k}=\left\{j\in I:\sum_{i\in I}I\left(\zeta_{j}\leq \zeta_{i}\right)\leq k\right\}$;      16:       $\tilde{A}=\left(A\setminus A_{k}\right)⋃I_{k}$, $\tilde{I}=\left(I\setminus I_{k}\right)⋃A_{k}$, ${\tilde{d}}_{\tilde{I}}=X_{\tilde{I}}^{T}\left(Y-X_{\tilde{A}}\beta_{\tilde{A}}\right)$, ${\tilde{\beta }}_{\tilde{A}}={\left(X_{\tilde{A}}^{T}X_{\tilde{A}}\right)}^{-1}X_{\tilde{A}}^{T}Y$;      17:       Calculate Loss function $L_{n}=\frac{1}{2n}||Y-X_{\tilde{A}}{\tilde{\beta }}_{\tilde{A}}{\left|\right|}_{2}^{2}$;      18:       **if** $L_{n}<L$ **then**      19:          $\beta_{A}={\tilde{\beta }}_{\tilde{A}}$, $d_{I}={\tilde{d}}_{\tilde{I}}$, $A=\tilde{A}$, $I=\tilde{I}$, $L=L_{n}$;      20:       **end if**      21:    **end for**      22:**until** $L_{0}-L<\tau$      23:Merge active set A with inactive set I to generate a new individual *A*.      24:**Output:** A new individual *A*.

### 2.2. The Proposed Method

**Figure 2:**
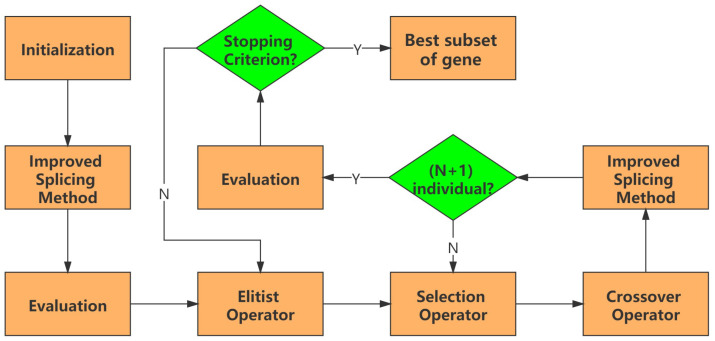
The flow chart of the proposed optimizer.

A modified memetic algorithm, genetic algorithm based on an improved splicing method, is a modified version of GA. To accelerate the search to identify the minimal best subset of genes, a new local search operator, improved splicing method, is utilized to improve starting points and mutation operator is replaced with the improved splicing method to enhance exploitation capability for achieving balance between exploitation and exploration of GA. In addition, the elitist operator is introduced into GA to prevent loss of the important subset of genes. The proposed algorithm is consisted of the following steps and the flow chart is shown in Figure 2.

**Step 1 Initialization:**

Set t=1 and Maximal number of generation *T*; *N* subsets of genes $a_{i}\left(t\right),i=1,\cdots ,N$, is randomly generated, consisted of a population, i.e., $P\left(t\right)=[a_{1}\left(t\right),\cdots ,a_{N}\left(t\right)]$. Each subset of genes is randomly generated in terms of Algorithm 1;

**Step 2 Improved Splicing Method:**

The improved splicing method shown in Algorithm 2 is used to improve each subset of genes $a_{i}\left(t\right)$ in P(t),i=1,2,⋯,N;

**Step 3 Evaluation:**

Calculate fitness value $F_{i}$ of each subset of genes $a_{i}\left(t\right),i=1,2,\cdots ,N$, in initial population P(t);

**Step 4 New Population:**

Set empty list of new population NP, i.e., NP=[];

**Step 5 Elitist Operator:**

The best subset of genes B(t) in the current generation is directly added into new the population NP, i.e., NP=[B(t),NP];

**Step 6 Selection Operator:**

Proportional roulette wheel selection is utilized to select parents $a_{i}\left(t\right)$ and $a_{j}\left(t\right)$ in the current population P(t);

**Step 7 Crossover Operator:**

Parents $a_{i}\left(t\right)$ and $a_{j}\left(t\right)$ are recombined to produce offspring $o_{i}\left(t\right)$ and $o_{j}\left(t\right)$ by a uniform crossover;

**Step 8 Improved Splicing Method:**

The improved splicing method, shown in Algorithm 1, is used to improve offspring $o_{i}\left(t\right)$ and $o_{j}\left(t\right)$. Then, improved offspring $o_{i}\left(t\right)$ and $o_{j}\left(t\right)$ are added into the new population NP, i.e., $NP=[o_{i}\left(t\right),o_{j}\left(t\right),NP]$;

**Step 9 Repeat:**

Repeat **Steps 6–8** until producing (N+1) individuals;

**Step 10 Evaluation:**

t=t+1; P(t)=NP in population of the next generation; calculate fitness value $F_{i}$ of each subset of genes $a_{i}\left(t\right),i=1,2,\cdots ,N$, in the new population P(t);

**Step 11 Stopping Criterion:**

Repeat **Steps 4–10** until maximum number of generations *T* is satisfied or no improvement over 5 generations continuously. Then, go to **Step 12**;

**Step 12 Output:**

Output the best subset of genes.

## 3. Materials

To check the performance of the proposed optimizer, seven well-known feature selection methods are compared with it, including adaptive best-subset selection (ABSS) [24], genetic algorithm (GA) [25], binary particle swarm optimization (BPSO) [30], binary salp swarm algorithm (BSWA) [27], sine cosine optimization algorithm (SCA) [29], artificial bee colony algorithm (ABC) [28], and β-hill climbing [26].

### 3.1. The Investigated Data

In meat production, the body weight (BW) of sheep is a key economic trail [35]. As pointed out by Cao et al. [36], BWs measured at birth and other life stages are major indicators for productivity, health, and preventive management. In genetics, some SNPs are associated with BW, and the identification of these SNPs can improve the efficiency of sheep breeding programs. However, selecting the significant SNPs among sheep genes is a NP-hard problem [37].

Here, to investigate the effectiveness of the proposed method, we conduct experiments on the dataset of three measures of body weights of 240 Hu sheep, including birth weight, six-month weight, and weaning weight. The dataset is available in the GEO accession number GSE152717 [36]. We exclude some genes containing missing values. Then, a brief dataset characteristic for three types of body weight of Hu sheep is shown in Table 1.

### 3.2. The Fitness Function Setting

The mean squared error (MSE) is used as fitness function in the paper, and support vector regression (SVR) [38] is used as a regression model to predict body weights at three different times in this paper.

### 3.3. The Hyper-Parameter Setting

**Figure 3:**
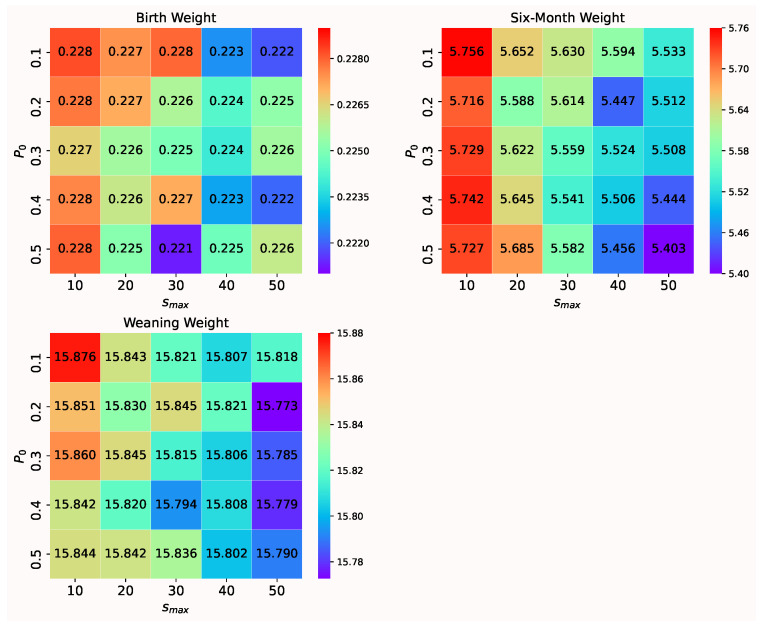
Mean squared error with different combinations $s_{max}$ and $P_{0}$ for analyzing the body weights on three occasions.

For the body weight measured on each occasion, the hyper-parameters of the proposed method are set as follows: Number of generations T=30; Number of individuals N=20; Threshold τ=0.01|A|log(p)log(logn)/n [24], where |A| is the size of the active set, *n* is the number of training sets and *p* is the number of features; Parameters of SVR, including penalty parameter *C*, ϵ in ϵ-insensitive loss function and σ in Gaussian kernel function are shown in Table 2; The expected size of the subset of genes s=2000; For maximal size of the subset of genes $S_{max}$ and crossover disruption probability $P_{0}$, the grid search is utilize to search the optimal combination $S_{max}$ and $P_{0}$. Range of $P_{0}$ is set as {0.1,0.2,0.3,0.4,0.5} and range of $S_{max}$ is set as {10,20,30,40,50}. The result is shown in Figure 3. In the dataset of the birth weight, when $S_{max}=30,P_{0}=0.5$, MSE is smaller; In the dataset of the six-month weight, when $S_{max}=40,P_{0}=0.5$, MSE is smaller; In the dataset of the weaning weight, when $S_{max}=50,P_{0}=0.2$, MSE is smaller. The other parameter settings of the proposed method for the body weights on each occasion are reported in Table 2. Additionally, the parameter settings for the considered benchmark methods are recorded in Appendix A.

## 4. Results and Discussion

To test the performance of feature selection methods, for predicting the body weights on each occasion, the instances are divided into training set (170 samples) and test set (70 samples). The training set is used to obtain the relevant subset of genes while the test set is used to evaluate MSE of SVR [39]. The initialization of individuals uses Algorithm 1, the fitness function uses MSE, and the regression model uses SVR in the other heuristic methods for fair comparison. The experiment results are shown in Table 3. Some main points can be obtained from our experiment as follows.

### 4.1. SVR vs. Other Heuristic Methods

In Table 3, the SVR models with heuristic methods, including β-Hill Climbing, SWA, ABC, SCA, BPSO, can significantly reduce the candidatures of gene to nearly 2000 genes. Furthermore, according to the error indicator (MSE), the performance of these SVR models is very similar. For example, all indicators for the birth weight are 0.239, thus we can confirm that more than 52,000 genes are irrelevant to the birth weight.

### 4.2. Proposed Method vs. ABSS

In Table 3, the performance of SVR combined with the proposed method is more outstanding compared with SVR combined with ABSS for the body weight of the Hu sheep on each occasion. For the six-month weight, the number of selected genes is only a few by ABSS, and the performance of SVR significantly worsens. This means that ABSS may be limited by correlation feature method in the initial step of ABSS. It cannot provide the promising subset of genes of the entire gene subset space while the proposed method can provide the promising subset of genes of the entire gene subset space by selection and crossover operators.

### 4.3. Proposed Method vs. GA

**Figure 4:**
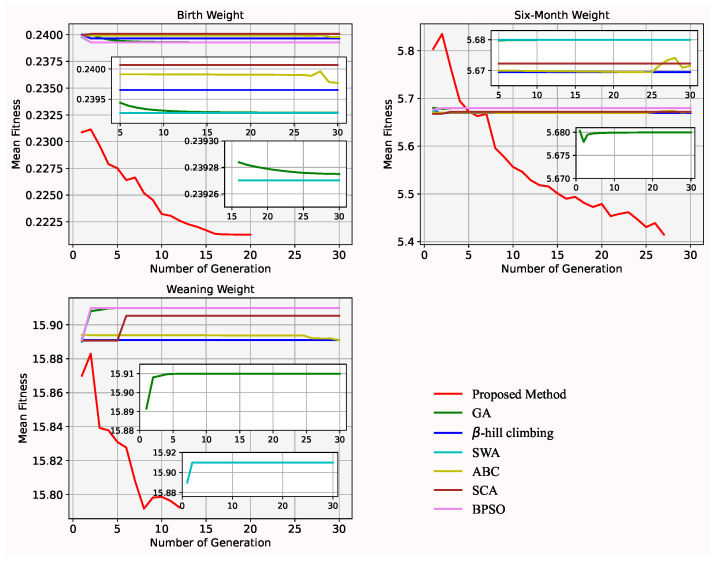
Mean fitness value of GA and the proposed method for the dataset of the body weights on three occasions.

**Figure 5:**
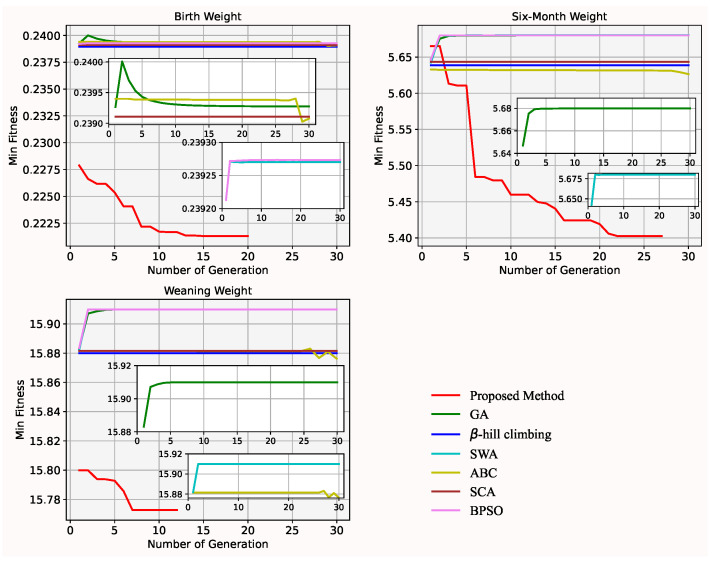
Min fitness value of GA and the proposed method for the body weights on each of the three occasions.

In Figure 4 and Figure 5, mean and min fitness value of each generation in the proposed method are less than in GA over 5 generations or more for the dataset of body weight at each of the three time points. Both the mean and min fitness value of each generation gradually decreases (in the proposed method) and increases (in GA) over generations for the body weight at 6 and 12 months, and at weaning, respectively. It shows that the mutation operator lacks exploitation capability while the improved splicing method has strong exploitation capability. In addition, in Figure 5, a minimal min fitness value is found in a few generations. It means that the initial start points, and offspring improved by the improved splicing method, can enhance the efficiency of search. Furthermore, in Table 3, the quality of the selected subset of genes by the proposed method not only is better than by GA, but also the size of the selected gene subset by the proposed method is less than by GA. It shows that the improved splicing method is embedded into GA to find the minimal best size of subset of genes.

### 4.4. Proposed Method vs. Other Heuristic Algorithms

In Figure 4, mean fitness almost remain unchanged over 5 generations in other state-of-arts optimization algorithms while mean fitness almost continuously decreases over 5 generations in the proposed method. It shows that the improved splicing method can prevent premature convergence to identify best gene subsets while the other optimization algorithms easily encounter sub-optimal gene subsets. Furthermore, in Table 3, the performance of SVR combined with the proposed method outperforms SVR combined with other heuristic methods, including β-hill climbing, SWA, ABC, SCA, and BPSO. The size of the selected gene subset by the proposed method is smaller than by heuristic algorithm, including β-hill climbing, SWA, ABC, SCA, and BPSO. Here, we can conclude that the proposed method is a successful heuristic algorithm to find the minimal best subset of genes for gene selection problems.

### 4.5. Selected Genes

**Figure 6:**
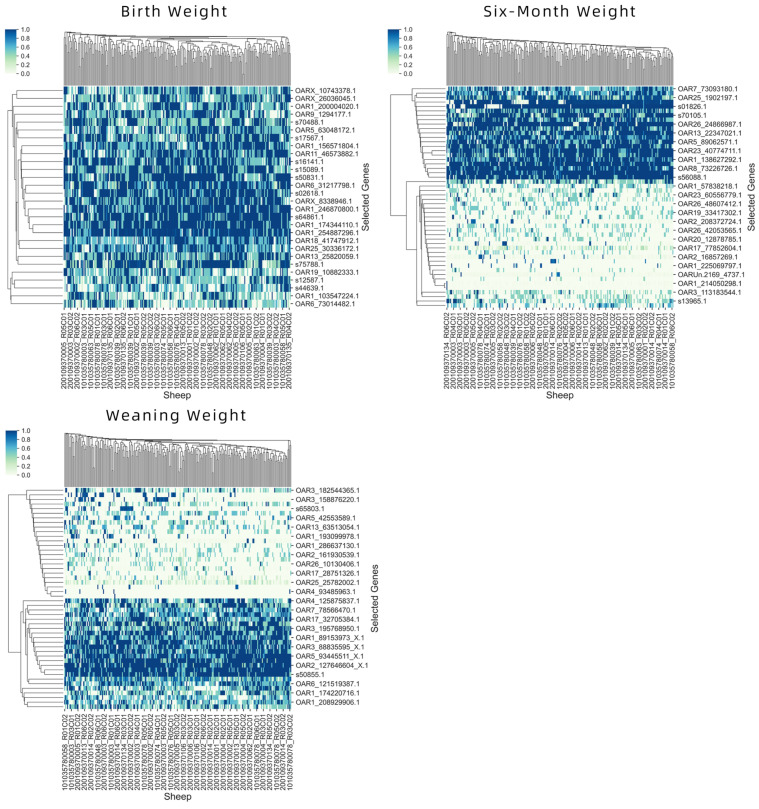
The heatmap of the actual expression profiles for the best subset of genes obtained from the proposed method.

The experimental results exhibit that the proposed method can yield minimal best gene subsets compared with other feature selections. Thus, Table 4 shows the best subset of genes obtained by using the proposed method for the body weight on the three occasions. Figure 6 displays a heatmap created for the identified best subset of genes for the body weight at the three occasions. The heatmap describe degree of similar and dissimilar among selected genes and sheep.

### 4.6. Statistical Analysis

The non-parametric Friedman test is used to show whether there exists any statistically significant difference among 8 feature selection methods. The average rank of each algorithm is shown in Table 5. As shown in Table 5, the proposed method has placed in rank one. The Iman and Davenport statistic $F_{F}$ [40] is calculated as 5.579. The result is much larger than critical values ($F_{0.1}\left(7,14\right)=2.19$). This means that the null hypothesis is rejected, i.e., there are significant differences among the eight feature selection methods. Then, the Nemenyi test in post hoc Holm test [40] further is employed to show significant difference between the proposed method and other feature selection methods. As shown in Table 6, there are significant difference between the proposed method and GA and the proposed method and BPSO.

## 5. Conclusions

In this paper, a modified memetic algorithm, a genetic algorithm based on an improved splicing method, has been proposed for gene selection problems. Different from traditional genetic algorithm, the optimizer can accelerate search to identify the minimal best subset of genes. It can absorb characteristics of crossover and selection operators to provide the promising subset of genes of the entire gene subset space. Furthermore, the improved splicing method can reduce the size of the promising subset of gene and recover the true subset of genes with a high probability. The initial points are improved by the improved splicing method to enhance efficiency of search, and the mutation operator is replaced by the improved splicing method to enhance exploitation capability for achieving balance between exploration and exploitation of GA. Therefore, the proposed optimizer can effectively achieve the best minimal subset of genes out of thousands of genes. Moreover, by using the body weights on each of the three occasions, we have demonstrated that our modified memetic algorithm can find the best minimal subset of genes compared with all considered algorithms, including ABSS. In addition, the proposed optimizer can be generalized to other high dimensional optimization problems [41,42]. 

## Figures and Tables

**Table 1:** The characteristics of a dataset for three measures of BWs from Hu sheep.

Type of BW	Number of Genes	Number of Instances
Birth weight	54,183	240
Six-month weight	54,183	240
Weaning weight	54,183	240

**Table 2:** The parameters values used in the proposed method for analyzing the body weights.

Type of BW	$S_{\max}$	$P_{0}$	C	ϵ	σ	T	N	s
Birth Weight	30	0.5	0.1	0.01	$10^{-5}$	30	20	2000
Six-Month Weight	50	0.5	1	0.01	0.01	30	20	2000
Weaning Weight	50	0.2	0.1	0.01	$10^{-5}$	30	20	2000

**Table 3:** Performance of feature selection methods for predicting the body weights on three occasions.

	Birth Weight		Six-Month Weight		Weaning Weight	
Method	MSE	NumF	MSE	NumF	MSE	NumF
SVR	0.2393	All	5.6800	All	15.9099	All
SWA	0.2392	2053	5.6424	1947	15.8819	2018
ABC	0.2390	1890	5.6264	2025	15.8762	1947
SCA	0.2391	1954	5.6433	2022	15.8815	1964
GA	0.2393	1995	5.6470	1905	15.8831	1952
BPSO	0.2392	1923	5.6464	2080	15.8835	1922
β-hill climbing	0.2389	1928	5.6385	1995	15.8799	1984
ABSS	0.2292	9	5.8182	9	15.8993	9
**Proposed method**	**0.2213**	**28**	**5.4026**	**50**	**15.7727**	**48**

Note: NumF-number of feature.

**Table 4:** Selected genes by using the proposed method for the body weight on the three occasions.

Type of BW	Selected Genes
Birth Weight	OAR1_103547224.1,OAR1_156571804.1,OAR1_174344110.1, OAR1_200004020.1,OAR1_246870800.1,OAR1_254887296.1, OAR11_46573882.1,OAR13_25820059.1,OAR18_41747912.1, OAR19_10882333.1,OAR25_30336172.1,OAR5_63048172.1, OAR6_31217798.1,OAR6_73014482.1,OAR9_1294177.1, OARX_10743378.1,OARX_26036045.1,OARX_8338946.1, s02618.1,s12587.1,s15089.1,s16141.1,s17567.1, s44639.1,s50831.1,s64861.1,s70488.1,s75788.1
Six-Month Weight	OAR1_103051402.1,OAR1_138627292.1,OAR1_214050298.1, OAR1_225069797.1,OAR1_252270534.1,OAR1_57838218.1, OAR1_72149006.1,OAR11_43264793_X.1,OAR13_22347021.1, OAR13_9894722.1,OAR16_60244426.1,OAR17_77852604.1, OAR19_33417302.1,OAR2_149404956.1,OAR2_16857269.1, OAR2_208372724.1,OAR20_12878785.1,OAR21_26401940.1, OAR23_40774711.1,OAR23_60556779.1,OAR25_1902197.1, OAR25_41478486.1,OAR26_24866987.1,OAR26_42053565.1, OAR26_48607412.1,OAR3_113183544.1,OAR3_119620209.1, OAR3_27184388.1,OAR3_88091256.1,OAR5_89062571.1, OAR7_73093180.1,OAR7_97719696.1,OAR8_73226726.1, OARUn.2169_4737.1,s01688.1,s01826.1,s06354.1, s07270.1,s13965.1,s14962.1,s17349.1,s35998.1, s36469.1,s52321.1,s56088.1,s64103.1,s70105.1, s71447.1,s72138.1,s72816.1
Weaning Weight	OAR1_174220716.1,OAR1_193099978.1,OAR1_208929906.1, OAR1_285395930.1,OAR1_286637130.1,OAR1_89153973_X.1, OAR11_8041122.1,OAR13_58349162.1,OAR13_63513054.1, OAR16_67669492.1,OAR17_12809597.1,OAR17_28751326.1, OAR17_32705384.1,OAR17_37807906.1,OAR18_55245057.1, OAR19_41399545.1,OAR2_127646604_X.1,OAR2_130068033.1, OAR2_161930539.1,OAR2_75375830_X.1,OAR20_5632451.1, OAR22_28398167.1,OAR25_25782002.1,OAR26_10130406.1, OAR3_158876220.1,OAR3_182544365.1,OAR3_195768950.1, OAR3_235746854.1,OAR3_88835595_X.1,OAR4_125875837.1, OAR4_93485963.1,OAR4_97984717.1,OAR5_42553589.1, OAR5_93445511_X.1,OAR6_121519387.1,OAR7_78566470.1, OAR8_36682621.1,OAR9_31965185.1,s07941.1,s26017.1, s44731.1,s48924.1,s50855.1,s56042.1,s56962.1, s59822.1,s65507.1,s65803.1

**Table 5:** Average rankings of MSE among 8 algorithms on three datasets using Friedman test.

	Proposed	SWA	ABC	SCA	GA	BPSO	β-Hill Climbing	ABSS
Rank	1	5.17	2.67	4.67	7	6.5	3	6

**Table 6:** Post hoc Holm test (0.1).

Comparison	*p*-Values	Result
Proposed vs. SWA	0.428	$H_{0}$ is not rejected
Proposed vs. ABC	0.900	$H_{0}$ is not rejected
Proposed vs. SCA	0.583	$H_{0}$ is not rejected
Proposed vs. GA	0.055	$H_{0}$ is rejected
Proposed vs. BPSO	0.108	$H_{0}$ is rejected
Proposed vs. β-Hill Climbing	0.900	$H_{0}$ is not rejected
Proposed vs. ABSS	0.195	$H_{0}$ is not rejected

## Data Availability

The data used to support the findings of this study are available from Wang, Y.-G. upon reasonable request.

## Appendix A

The parameter settings of SVR for analyzing the body weights are set as follows: In Birth weight, C=0.1, ϵ=0.01, $\sigma =10^{-5}$; In six-month weight, C=1, ϵ=0.01, σ=0.01; In weaning weight, C=0.1, ϵ=0.01, $\sigma =10^{-5}$; crossover disruption probability $P_{0}$ settings of GA for the body weights are set as follows: $P_{0}=0.5$ in birth weight; $P_{0}=0.5$ in six-month weight; $P_{0}=0.2$ in weaning weight. The parameters settings of other feature selection methods for the body weights are reported in Table A1.

**Table A1:** The parameters settings of other feature selection methods in analyzing the body weights.

Method	Parameter
GA [25]	T=30, N=20, s=2000, $p_{m}=0.047$
BPSO [30]	T=30, N=20, s=2000, $v_{max}=6$, w=0.7298, $c_{1}=1.49618$, $c_{2}=1.49618$
SCA [29]	T=30, N=20, s=2000, α=1
ABC [28]	T=30, N=20, s=2000, Trials limit=50, Flips limit=0.1
SWA [27]	T=30, N=20, s=2000
β-hill climbing [26]	T=30, N=20, s=2000, N=0.9, β=0.5
ABSS [24]	τ=0.01|A|log(p)log(logn)/n, $s_{max}=\left[\frac{n}{\log p\log(\log n)}\right]$, $k_{max}=\left|A\right|$
